# Feral cats do not play a major role in leptospirosis epidemiology on Reunion Island

**DOI:** 10.1017/S0950268819000190

**Published:** 2019-02-22

**Authors:** Y. Gomard, E. Lagadec, L. Humeau, P. Pinet, S. Bureau, D. Da Silva, M. Turpin, Y. Soulaimana Mattoir, S. Georger, P. Mavingui, P. Tortosa

**Affiliations:** 1 Université de La Réunion, UMR PIMIT (Processus Infectieux en Milieu Insulaire Tropical), INSERM 1187, CNRS 9192, IRD 249, Plateforme Technologique CYROI, Sainte-Clotilde, La Réunion, France; 2 Université de La Réunion, UMR PVBMT (Peuplements Végétaux et Bioagresseurs en Milieu Tropical), CIRAD, CS 92003, 97744 Saint Denis Cedex 9, La Réunion, France; 3 Parc National de La Réunion, LIFE+ Pétrels, 258 Rue de la République, 97431, Plaine des Palmistes, La Réunion, France; 4 Université de La Réunion, UMR ENTROPIE (Ecologie Marine Tropicale des Océans Pacifique et Indien), IRD, CNRS, CS 92003, 97744 Saint Denis Cedex 9, La Réunion, France; 5 Société d’Études Ornithologiques de La Réunion, 3 ruelle des Orchidées, Cambuston, 97440 Saint-André, La Réunion, France; 6 Association de Valorisation de l'Entre-Deux monde (AVE2M), 13 rue Josémont-Lauret /PK27, Bourg-Murat, 97418 Plaine des Cafres, La Réunion, France; 7 Ecologie Microbienne UMR CNRS 5557, Université Lyon 1, Villeurbanne, France

**Keywords:** Feral cats, *Leptospira*, renal carriage, Reunion Island

## Abstract

Although previous studies have reported *Leptospira* carriage in kidneys and urine of cats, the role of these animals in leptospirosis epidemiology remains poorly understood. Using molecular methods, we investigated *Leptospira* renal carriage in 172 feral cats from Reunion Island, an oceanic geographically isolated island located in the South West Indian Ocean. Only one out of the 172 analysed specimens tested positive for *Leptospira* DNA through quantitative real-time polymerase chain reaction. Using this positive sample, we could obtain sequences at three *Leptospira loci* (*rrs2*, *lipL32* and *lipL41*) allowing to report for the first time *Leptospira borgpetersenii* naturally infecting cats. Comparisons with bacterial sequences from both acute human cases and animal reservoirs revealed similarities with *Leptospira* sequences previously reported on Reunion Island. However, the low prevalence (0.6%) reported herein does not support any major role of feral cats in leptospirosis epidemiology on Reunion Island, contrasting with results recently reported on another Indian Ocean Island, Christmas Island. The significance of these discrepancies is discussed.

Leptospirosis is a widespread re-emerging infectious disease caused by pathogenic bacteria belonging to the genus *Leptospira* (Spirochaetales, Leptospiraceae) [1]. Pathogenic *Leptospira* are maintained in the renal tubules of animal reservoirs, which contaminate the environment through their urine. Human infection occurs either through contact with the animal's urine or contaminated environment [1]. It is estimated that leptospirosis causes over 1 million human cases per year, leading to nearly 60 000 fatal cases [2]. The disease incidence is higher in subtropical regions [3] probably due to environmental conditions (increased humidity and temperature) favourable to *Leptospira* maintenance and transmission.

Leptospirosis represents a major burden in the South West Indian Ocean (SWIO) region, with some islands such as Seychelles displaying amongst the highest incidence worldwide [3, 4]. In this context, considerable efforts have been made to characterise leptospirosis epidemiology in the region. Recently, molecular studies comparing *Leptospira* sequence types obtained from acute human cases and animal reservoirs have identified a number of probable important reservoirs [4–7]. These studies have shown that beside rats, other animals play a significant role in *Leptospira* epidemiology including tenrecs, a family of small insectivorous mammals endemic to Madagascar, as well as introduced mammals such as cows, mice and dogs [4, 6, 7]. However, for some *Leptospira* lineages infecting humans, the animal reservoir(s) still remain(s) to be identified.

The reported presence of pathogenic leptospiral DNA in the urine of cats [6, 8, 9] strengthens the need for an investigation of this potential reservoir. *Leptospira* carriage has been previously reported in stray cats on Reunion Island [10] although no sequences were produced and hence precluded any molecular comparison with bacterial strains characterised from human acute cases and animals. Recently, feral cats have been reported as important carriers of pathogenic *Leptospira* on Christmas Island [11], a true oceanic Island located in the Eastern Indian Ocean. These data stimulate the need for investigating feral cats as reservoirs of pathogenic *Leptospira* on Reunion Island.

This study was carried out in the frame of the LIFE+ Pétrels project (http://www.petrels.re), a conservation project aiming at protecting two endemic and endangered seabird species of Reunion Island (55°39′E, 21°00′S), namely Barau's Petrel (*Pterodroma baraui*) and Mascarene Petrel (*Pseudobulweria aterrima*). Feral cats are known to predate the Barau's Petrel (eggs, chicks, juveniles and adults) [12] and removal of feral cats in nesting areas is a major conservation action implemented locally by the LIFE+ Pétrels partners in collaboration with a local non-governmental organisation called AVE2M. The protocols of research were approved by the CYROI institutional ethical committee, certified by the French Ministry of Higher Education and Research (NoAPAFIS#6916-20151 00213267087 v6). Cats were trapped between July 2015 and December 2016 using live traps at six different sites from 110 to 2850 m elevation, in disturbed and preserved areas. The periods of sampling covered the two local seasons: cool–dry season from July to October and hot–wet summer from November to June. The animals were then euthanised by the veterinary clinic of Saint Louis. Different tissues samples were taken (heart, stomachs, kidneys and blood) and stored at −80 °C until laboratory analyses.

For each animal, total nucleic acids were extracted from a small piece of kidney using the DNeasy Blood and Tissue kit (Qiagen) following the manufacturer's recommendations. *Leptospira* detection was performed on each DNA extract by using a specific protocol of quantitative real-time polymerase chain reaction (qPCR) targeting the 16S gene (*rrs2*) of pathogenic *Leptospira* [13]. On each qPCR-positive sample, *Leptospira* was genotyped using a multilocus sequence typing (MLST) (pubmlst.org, scheme# 3) encompassing six genes (*secY*, *adk*, *rrs2*, *icdA*, *lipL32* and *lipL41*) [14] and optimised in order to characterise the *Leptospira* diversity actually circulating in the SWIO region [15]. Each PCR product was visualised under UV light after migration on a 2% agarose gel containing 1X GelRed™ (Biotum Inc., Hayward, CA, USA). The PCR products were sequenced on both strands (Genoscreen, Lille, France) by using the corresponding set of primers and all sequences were deposited in GenBank (MH820176–MH820178).

A total of 172 feral cats were tested for pathogenic *Leptospira* renal carriage of which only one tested positive (cycle threshold for qPCR: 37.6) yielding a prevalence of 0.6%. The single positive animal corresponded to an adult female sampled in a disturbed mountain rainforest. Full MLST was attempted on this sample but a sequence could be obtained for only three out of the six MLST *loci*, *rrs2*, *lipL32* and *lipL41*, showing that the infecting *Leptospira* corresponded to *Leptospira borgpetersenii*. The comparison of the sequences with the PubMLST database (pubmlst.org, scheme# 3) indicates that *rrs2* and *lipL32* sequences are new and closely related to the *rrs2* allele 20 (one nucleotide difference) and *lipL32* allele 30 (one nucleotide difference), respectively, while the *lipL41* sequence corresponds to *lipL41* allele 38. Although full genotyping could not be achieved, the combination of these three alleles indicates that the detected *L. borgpetersenii* could correspond to the Sequence Type 127. We then compared each allele to sequences previously obtained from acute human cases and wild mammals on Reunion Island. Interestingly, *rrs2* sequence (503 bp) showed 100% identity with a sequence previously obtained from a human case on Reunion Island (*Homo sapiens* #213013106601; GenBank number: KU183592). The *lipL32* sequence (434 bp) showed 100% identity with the *lipL32* sequence obtained from this same human case (*H. sapiens* #213013106601; GenBank number KU183575) and also from a house mouse (*Mus musculus* BLA030; GenBank number: KU183573). The *lipL41* sequence (411 bp) showed 100% identity with a sequence reported from another human case (*H. sapiens* #31658, GenBank number: KU183581).

This study confirms the presence of pathogenic *Leptospira* infection in cats from Reunion Island [6, 10]. However, the detected prevalence is extremely low (0.6%, 1/172) as compared with that previously reported on stray cats from Reunion Island (28.6%, 6/21) [10] or from the neighbouring Seychelles (8.3%, 1/12) [4]. Feral and stray cats occupy distinct ecological niches and it is possible that the difference in infection prevalence results from distinct levels of exposure to pathogenic *Leptospira* (i.e. differences of prey and/or environments). The study of Dybing *et al*. [11] reported high infection prevalence in feral cats on the tropical Christmas Island, but the absence of renal carriage in feral cats from other territories with arid or temperate environments (Dirk Hartog Island and southwest Western Australia, respectively). The authors proposed that the tropical climate on Christmas Island is favourable to survival and hence transmission of the bacteria [11]. In the present study, although evolving in tropical conditions, Reunion feral cats display low renal carriage throughout the year. These differences among islands are in keeping with other studies showing distinct transmission chains (*i.e.* reservoirs and *Leptospira* species) in different islands within the same region [4, 6, 7] and stimulate a thorough investigation of the epidemiology in each environmental setup.

The implication of cats in leptospirosis epidemiology remains poorly investigated and most of the available studies are based on serological approaches (Microscopic Agglutination Test) (see in [16]), which bring in information on the actual exposure of animals to *Leptospira* but certainly not on their role as a biological reservoir. Indeed, studies reported the absence of congruence between serology and PCR results in different mammal species which indicated that the serology is not a relevant tool to predict the reservoir status of given species [8, 17, 18]. To our knowledge, only scarce molecular identification of *Leptospira* infecting cats is available, with two *Leptospira* species being previously reported, namely *Leptospira interrogans* and *Leptospira kirschneri* [8, 11]. We report for the first time *L. borgpetersenii* naturally infecting cats. On Reunion Island, *L. borgpetersenii* has been reported in cows and mice and rarely in human acute cases [6]. The detection of an identical *lipL32* sequence in the single positive cat and in a house mouse, which is typically a cat prey, supports a previously proposed hypothesis of infection through predation [19]. However, our data do not allow fully addressing such hypothesis, which would require gathering additional molecular data, including access to full genomes.

Altogether, the presence of pathogenic leptospiral DNA in the urine of cats confirms previous studies suggesting that these animals are a potential source of contamination for humans. However, more investigations are necessary to determine the epidemiological importance of this reservoir in the disease, including the isolation of *Leptospira* from cats’ urines. In the context of Reunion Island, although we detected identical *Leptospira* sequences in the single positive cat and in acute human cases on three *loci*, the low prevalence allows us rejecting any major role of feral cats in the local epidemiology of leptospirosis. These results are strikingly different from those reported on Christmas Island, where the prevalence is indeed high in cats [11]. Hence, this work supports previous published studies showing distinct transmission chains in the different islands of the SWIO region [4, 6, 7]. Altogether, these data pinpoint the importance of a proper molecular assessment of *Leptospira* prevailing at each specific environment in order to establish the role of major involved biological compartments and optimise the design of preventive measures.
